# Understanding the role of active site residues in CotB2 catalysis using a cluster model

**DOI:** 10.3762/bjoc.16.7

**Published:** 2020-01-08

**Authors:** Keren Raz, Ronja Driller, Thomas Brück, Bernhard Loll, Dan T Major

**Affiliations:** 1 Department of Chemistry, Bar-Ilan University, Ramat-Gan 52900, Israel; 2 Institute of Chemistry and Biochemistry, Laboratory of Structural Biochemistry, Freie Universität Berlin, Takustr. 6, 14195 Berlin, Germany; 3 present address: Department of Molecular Biology and Genetics, Aarhus University, Gustav Wieds Vej 10, 8000 Aarhus C, Denmark; 4 present address: Danish Research Institute of Translational Neuroscience - DANDRITE, Nordic-EMBL Partnership for Molecular Medicine, Aarhus C, Denmark; 5 Werner Siemens Chair of Synthetic Biotechnology, Dept. of Chemistry, Technical University of Munich (TUM), Lichtenbergstr. 4, 85748 Garching, Germany

**Keywords:** active site, CotB2 cyclase, diterpene, mechanism, quantum mechanics

## Abstract

Terpene cyclases are responsible for the initial cyclization cascade in the multistep synthesis of a large number of terpenes. CotB2 is a diterpene cyclase from *Streptomyces melanosporofaciens,* which catalyzes the formation of cycloocta-9-en-7-ol, a precursor to the next-generation anti-inflammatory drug cyclooctatin. In this work, we present evidence for the significant role of the active site's residues in CotB2 on the reaction energetics using quantum mechanical calculations in an active site cluster model. The results revealed the significant effect of the active site residues on the relative electronic energy of the intermediates and transition state structures with respect to gas phase data. A detailed understanding of the role of the enzyme environment on the CotB2 reaction cascade can provide important information towards a biosynthetic strategy for cyclooctatin and the biomanufacturing of related terpene structures.

## Introduction

Enzymes catalyze numerous complex biochemical reactions in different cellular compartments [1–2]. More specifically, the enigmatic class of terpene cyclases is responsible for converting linear aliphatic oligoprenyl diphosphates into various chemically complex macrocyclic products. The resulting terpene scaffolds and their functionalized terpenoid analogues comprise the largest and structurally most diverse family of natural products, currently representing over 80,000 reported structures from all kingdoms of life [3]. The largest diversity of terpenoids is reported for the plant kingdom where higher terpenes represent secondary metabolic products, which are responsible for, e.g., defense against biotic and abiotic stress or for attracting insects for pollination [4–5]. Industrially, terpene natural products are employed as flavoring agents [6], fragrances, pigments, cosmetics, perfumes, biofuels, and agrochemicals [5]. Additionally, terpene natural products with numerous pharmacological [7–8] and biological activities have been reported, rendering them important targets for medical and biotechnology research [9]. Chemical synthetic and sustainable biosynthetic strategies in synergy with the biological activity of different terpene natural products have been reviewed elsewhere [3,10–17].

The first crystal structure of a monoterpene cyclase [18] was reported in 2002. Subsequently, the first crystal structures of a sesquiterpene [19–20] and a triterpene [21] cyclase were published in 1997. Less than a decade ago, the first crystal structure of a diterpene cyclase was reported by Christianson and co-workers [22]. These structures, in conjunction with extensive biochemical work [10,13–14,23], have contributed to the understanding of mechanistic details of terpene cyclases and facilitated rational enzyme design [24]. Theoretical quantum mechanical (QM) investigations on the chemistry of terpenes in the gas phase have provided a detailed understanding of the carbocation mechanisms underlying terpene synthase function [25–27]. Further, we have used multiscale modeling tools to study the effects of the enzyme environment in catalyzing reactions of mono-, sesqui-, and diterpene synthases [28–36].

Diterpenes are generated from the universal aliphatic substrate geranyl geranyl pyrophosphate (GGPP) [4]. In vitro experiments demonstrated that many diterpenes have pharmaceutical applications by featuring anticancer, antibacterial, anti-inflammatory, and antiretroviral activities [37]. Moreover, they are applied in the food industry as antioxidants and sweeteners [4].

CotB2 is a bacterial diterpene cyclase from *S*. *melanosporofaciens,* which catalyzes the formation of cyclooctat-9-en-7-ol, representing the first committed step in the biosynthesis of the next-generation anti-inflammatory drug cyclooctatin. The intracellular target of cyclooctatin is an as of yet uncharacterized lysophospholipase, which is involved in early steps of the inflammatory signaling cascade [38–40]. In the last decade, numerous interdisciplinary studies have addressed the chemical mechanism of CotB2 catalysis utilizing different detection and analysis methods.

**Scheme 1:**
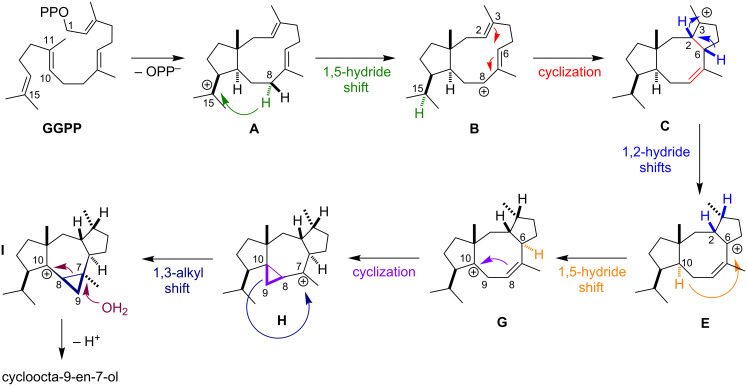
Mechanism for formation of cyclooctat-9-en-7-ol, published similarly in [42].

Meguro and co-workers [41] established the chemical mechanism for the formation of cyclooctatin using isotope labeling experiments (Scheme 1). Recently, Hong and Tantillo [38] and Sato and co-workers [39] investigated the CotB2 mechanism using QM tools. According to Meguro and co-workers [41], the cyclization process commences with the dissociation of the pyrophosphate leaving group of GGPP, forming an allylic carbocation, and two subsequent electrophilic cyclizations to generate intermediate **A**. Intermediate **A** undergoes a 1,5-hydride shift, forming intermediate **B**. A subsequent cyclization forms intermediate **C**. Intermediate **C** generates intermediate **E** via one of two possible pathways: either a direct 1,3-hydride shift or an indirect pathway involving two 1,2-hydride shifts. Theoretical investigations by Hong and Tantillo suggested that the indirect transformation from intermediate **C** to **E** is energetically favored and might be biosynthetically relevant [38]. This finding is in agreement with the report by Sato and co-workers [39], who performed isotope labeling experiments combined with QM calculations. Intermediate **G** forms via a 1,5-hydride shift from C6 to C10 to generate a homoallylic cation, and the formation of intermediate **H** occurs due to cyclization to yield a cyclopropyl ring. Intermediate **I** forms due to isomeric formation of a cyclopropylcarbinyl cation, as shown by isotope labeling [41]. QM calculations support this unusual 1,3-alkyl shift that interconverts **H** and **I** [38–39]. Finally, the cyclopropyl ring opens by virtue of a nucleophilic water attack, and cyclooctat-9-en-7-ol is formed.

Although gas phase calculations shed light on the reactivity of the isolated species and provided crucial mechanistic insights, the biorelevant mechanism cannot be fully understood without taking into account the enzyme–solvent environment. A common problem when studying these enzymes is the lack of high-resolution crystal structures that are biologically relevant, i.e., that have a ligand bound in a reactive configuration and have a fully closed active site. Recently, a crystal structure of the CotB2 enzyme that met these criteria was published [42]. In the current work, we describe the crucial role of the amino acids in the active site on the reaction energetics using QM calculations in an active site cluster model. The active site cluster theozyme model [43–44] was constructed from the crystal structure coordinates of active site amino acids, which were presumed to stabilize the carbocations during the reaction cascade. Each reaction step's relevant species was optimized in the active site model within a fixed enzyme approximation. The results obtained using the active site model were compared with gas phase data.

## Results and Discussion

**Figure 1:**
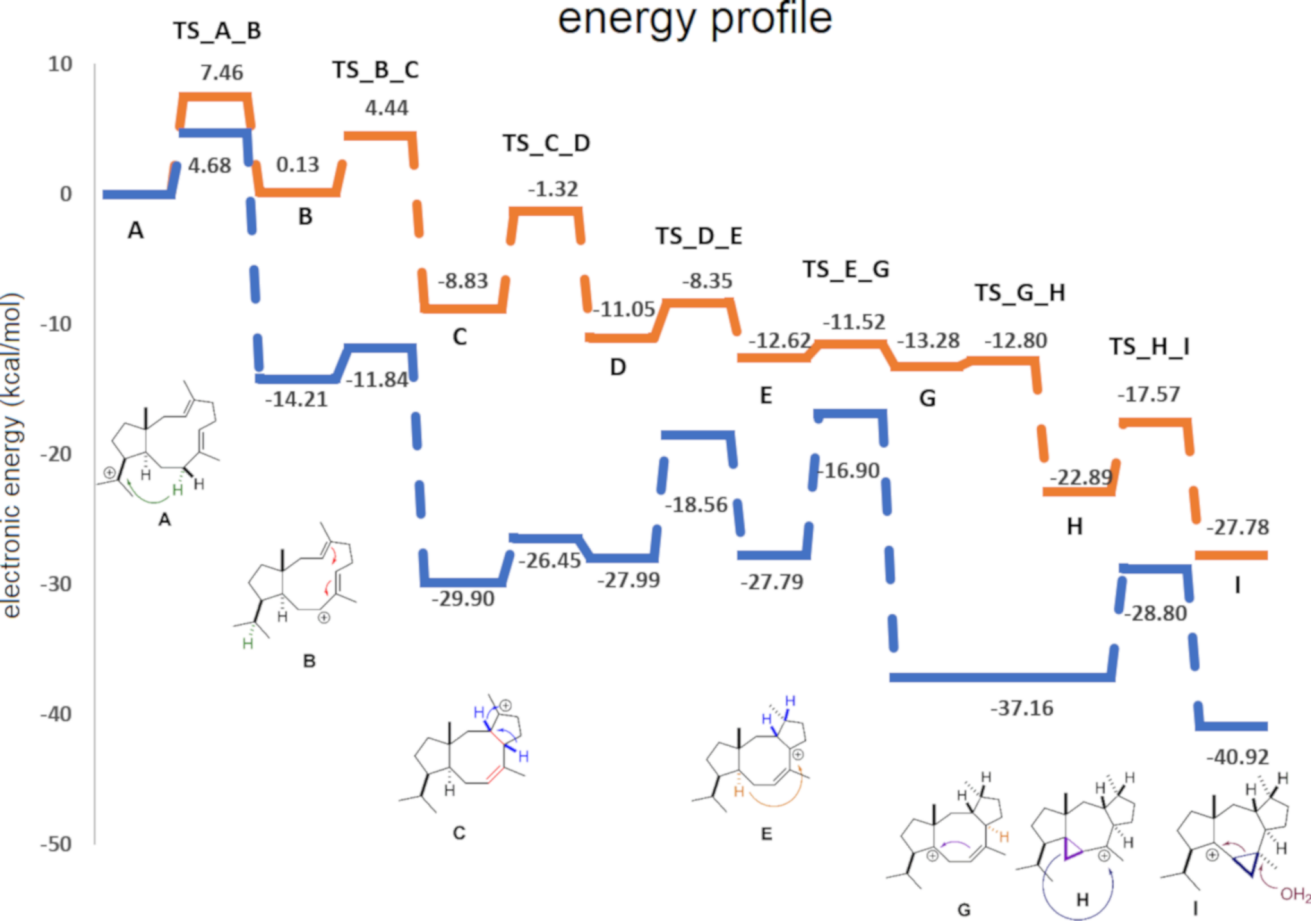
Computed electronic energy profiles (kcal/mol) for the CotB2 cyclase mechanism. The calculations used M062X/6-31+G(d,p).

The energy profiles for both gas phase (orange) and for the active site model (blue) reactions were characterized by a sequential decreasing pattern (Figure 1). The inspection of the gas phase profile revealed important information regarding the inherent reactivity [27] of the carbocation species. As the reaction proceeded, π-bonds transformed into σ-bonds, explaining the steady downhill progress of the energy profile. An additional feature was the relatively low energy barrier of less than ca. 10 kcal/mol separating the intermediates. The gas phase mechanism has been discussed extensively by Hong and Tantillo [38] and Sato and co-workers [39]. Herein, we focused on the differences between gas phase and active site model energies. All interaction distances are provided in Table 1, which provided the basis for the following categorization of interactions as π–cation, dipole–cation, and charge–cation. Note, that no attempts to quantify the individual pairwise interactions were made.

**Table 1:** Interactions between intermediates and TS structures with active site residues.

intermediate	interacting species		distance (Å)	interaction type

**A**	W186	C_15_	4.49	π-cation
	I181	C_15_	3.80	dipole-cation

**B**	N103	C_6_	4.66	dipole–cation (C=O)
	N103	C_7_	4.72	dipole–cation (C=O)
	N103	C_8_	4.41	dipole–cation (C=O)
	T106	C_6_	3.96	dipole–cation (OH)
	T106	C_7_	4.15	dipole–cation (OH)
	T106	C_8_	5.33	dipole–cation (OH)
	F107	C_6_	4.25	π–cation
	F107	C_7_	5.53	π–cation
	F107	C_8_	5.88	π–cation
	I181	C_6_	5.15	dipole–cation
	I181	C_7_	4.45	dipole–cation
	I181	C_8_	4.14	dipole–cation

**C**	O_3_	C_3_	4.20	anion–cation
	F107	C_3_	3.65	π–cation
	I181	C_3_	4.72	dipole–cation

**D**	O_3_	C_2_	5.03	anion–cation
	F107	C_2_	4.35	π–cation

**E**	N103	C_6_	4.74	dipole–cation (C=O)
	N103	C_7_	3.81	dipole–cation (C=O)
	N103	C_8_	3.04	dipole–cation (C=O)
	T106	C_6_	4.43	dipole–cation (OH)
	T106	C_7_	4.25	dipole–cation (OH)
	T106	C_8_	5.32	dipole–cation (OH)
	F107	C_6_	4.66	π–cation
	F107	C_7_	5.37	π–cation
	F107	C_8_	5.54	π–cation
	F149	C_6_	5.84	π–cation
	F149	C_7_	6.42	π–cation
	F149	C_8_	7.75	π-cation

**G**/**H**	N103	C_7_	5.91	dipole–cation
	T106	C_7_	5.27	dipole–cation (OH)
	F149	C_7_	5.35	π–cation
	I181	C_7_	3.08	dipole–cation (C=O)
	W186	C_7_	6.48	π–cation

**I**	N103	C_10_	5.44	dipole–cation
	I181	C_10_	3.31	dipole–cation (C=O)
	W186	C_10_	5.87	π–cation

TS structure	interaction species		distance Å	interaction type

**A**_**B**	I181	C_15_	3.85	dipole–cation
	I181	C_8_	4.80	dipole–cation
	W186	C_15_	4.48	π–cation
	W186	C_8_	5.09	π–cation

**B**_**C**	O_3_	C_2_	3.78	
	O_3_	C_6_	5.88	anion–cation
	N103	C_2_	6.26	
	N103	C_6_	4.80	dipole–cation (C=O)
	T106	C_2_	6.80	
	T106	C_6_	4.30	dipole–cation (OH)
	F107	C_2_	4.12	
	F107	C_6_	4.36	π–cation
	I181	C_2_	4.33	
	I181	C_6_	4.80	dipole–cation

**C**_**D**	O_3_	C_2_	4.88	anion–cation
	O_3_	C_3_	4.39	anion–cation
	F107	C_2_	4.42	π–cation
	F107	C_3_	3.61	π–cation
	I181	C_2_	4.00	dipole–cation
	I181	C_3_	4.85	dipole–cation

**D**_**E**	O_3_	C_2_	4.66	anion–cation
	O_3_	C_6_	5.82	anion–cation
	F107	C_2_	4.55	π–cation
	F107	C_6_	5.37	π–cation
	F149	C_2_	5.93	π–cation
	F149	C_6_	5.05	π–cation

**E**_**G**/**H**	N103	C_6_	6.09	dipole–cation (C=O)
	N103	C_10_	5.20	dipole–cation (C=O)
	F107	C_6_	5.07	π–cation
	F107	C_10_	5.15	π–cation
	F149	C_6_	5.42	π–cation
	I181	C_6_	3.62	dipole–cation (OH)
	I181	C_10_	3.84	dipole–cation (OH)

**G**/**H**_**I**	N103	C_7_	5.41	dipole–cation
	N103	C_10_	5.81	dipole–cation
	T106	C_7_	5.11	dipole–cation (OH)
	T106	C_10_	7.29	dipole–cation (OH)
	F149	C_7_	5.68	π–cation
	F149	C_10_	7.76	π–cation
	I181	C_7_	3.47	dipole–cation (C=O)
	I181	C_10_	3.03	dipole–cation (C=O)
	W186	C_7_	6.09	π–cation
	W186	C_10_	5.70	π–cation

**Figure 2:**
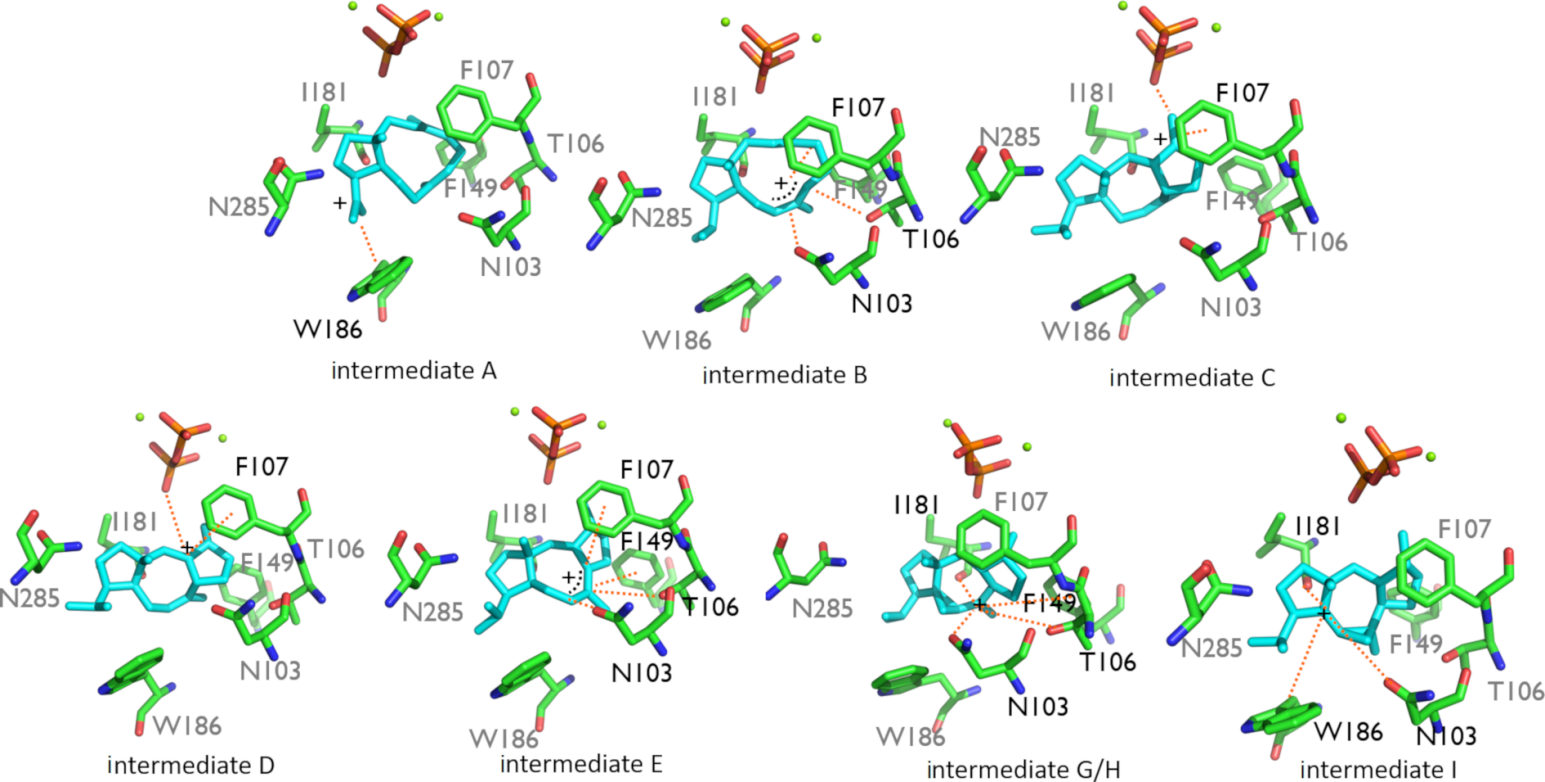
Intermediates **A**–**I** in the active site model. Interactions are marked by dashed orange lines, the interacting residues are labeled in black, the non-interacting residues are labeled in grey, and plus signs note location of the cations.

Carbocation **A** was stabilized through π–cation interaction with W186, while **B** was stabilized due to dipole–cation interactions of the allylic carbocation at C6–C7–C8 with N103, T106, and I181. These variations in interactions resulted in an energy difference of 14.2 kcal/mol, favoring **B**, and the barrier was reduced by 2.8 kcal/mol (Figure 1). Another possible reason for the stabilization was that C7 had a greater proximity to the pyrophosphate group than C15 (6.71 Å vs 7.54 Å, Table 1 and Figure 2). The energy difference between **B** and **C** was 15.7 kcal/mol in the active site model, compared to 8.7 kcal/mol in the gas phase. Here, the energy gain was likely due to the fact that the carbocation in intermediate **C** was located 4.21 Å away from the pyrophosphate group, which stabilized it (Table 1 and Figure 2). Moreover, π–cation interactions with F107 contributed to the stabilization as well. The activation energy for the formation of **C** was 2.4 kcal/mol in the active site model compared to 4.3 kcal/mol in the gas phase. In the active site model, **D** was less stable than **C** by almost 2 kcal/mol, while in the gas phase, **D** was more stable by ca. 2 kcal/mol. The main reason for this difference was possibly a difference in the conformation of **D** in the active site model compared to the gas phase. The dihedral angle defined by C3–C2–C6–C7 in **D** was greater by 53° in the active site model than in the gas phase, and the **D** dihedral angle C10–C9–C8–C7 in the active site was smaller by 258° than in the gas phase (Figure 4a). Moreover, the dihedral angle C2–C1–C11–C10 was greater by 281° in the active site model. The distance between C4 and C13 was significantly greater in the active site model (1.2 Å), indicating a more extended conformation. Figure 4a shows clearly that intermediate **D** was more folded in the gas phase than in the active site model. The required activation energy to form **D** was 4.1 kcal/mol lower in the active site model than the gas phase, likely due to π–cation interactions with F107 and F149 and greater proximity to the negatively charged pyrophosphate group. Another conformational difference between the gas phase and in the enzyme model was noted for **E** as well (Figure 4b). The dihedral angle C2–C3–C4–C5 was greater by 285°, and C10–C9–C8–C7 was smaller by 294° in the active site model than in the gas phase. Moreover, the angle C2–C1–C11 was greater by 5° in the active site model than in the gas phase, and the distance between C4 and C13 was smaller by 0.5 Å in the gas phase. The net result of these differences was that intermediate **E** was more folded in the gas phase, although it was not as dramatically folded as **D**. The reason for greater folding in the gas phase could have been a tendency to adopt conformations that maximized intramolecular dispersion interactions [45–46]. In the active site model of **E**, the carbocation at C6 had a greater distance from the pyrophosphate group than C2 in cation **D** (6.03 Å vs 5.03 Å) and likely contributed to a slight destabilizing effect in the active site model. This was in spite of interactions between cation **D** and N103, T106, F107, and F149. Nonetheless, the energy barrier to form **E** was higher in the active site model than in the gas phase. An elevated energy barrier was also observed for the formation of **G** (by almost 10 kcal/mol). This may be explained by the loss of interactions between **G** and the pyrophosphate moiety as the cation moved further away, deeper into the hydrophobic part of the pocket. A distinct carbocation **G** was not observed in the enzyme model. Instead, a cation resembling **H**, with a C8–C10 bond that was already partly formed, was observed. This carbocation was more stable in the active site model than **G** in the gas phase by almost 9 kcal/mol. Hence, in the enzyme model, cation **G** was not a stable species, and instead, **H** was formed spontaneously. The energy for the transformation of **E** to **H** was very similar in the gas phase and in the enzyme. Carbocation **H** formed interactions with N103, W186, and especially with I181. The relative energy difference between **H** and **I** was also similar in the gas phase and in the enzyme model. However, the activation energy was higher by 3.0 kcal/mol in the active site model, possibly due to steric effects. **I** was stabilized via interactions with N103, I181, and W186, which likely made similar stabilizing contributions as in cation **H**.

**Figure 3:**
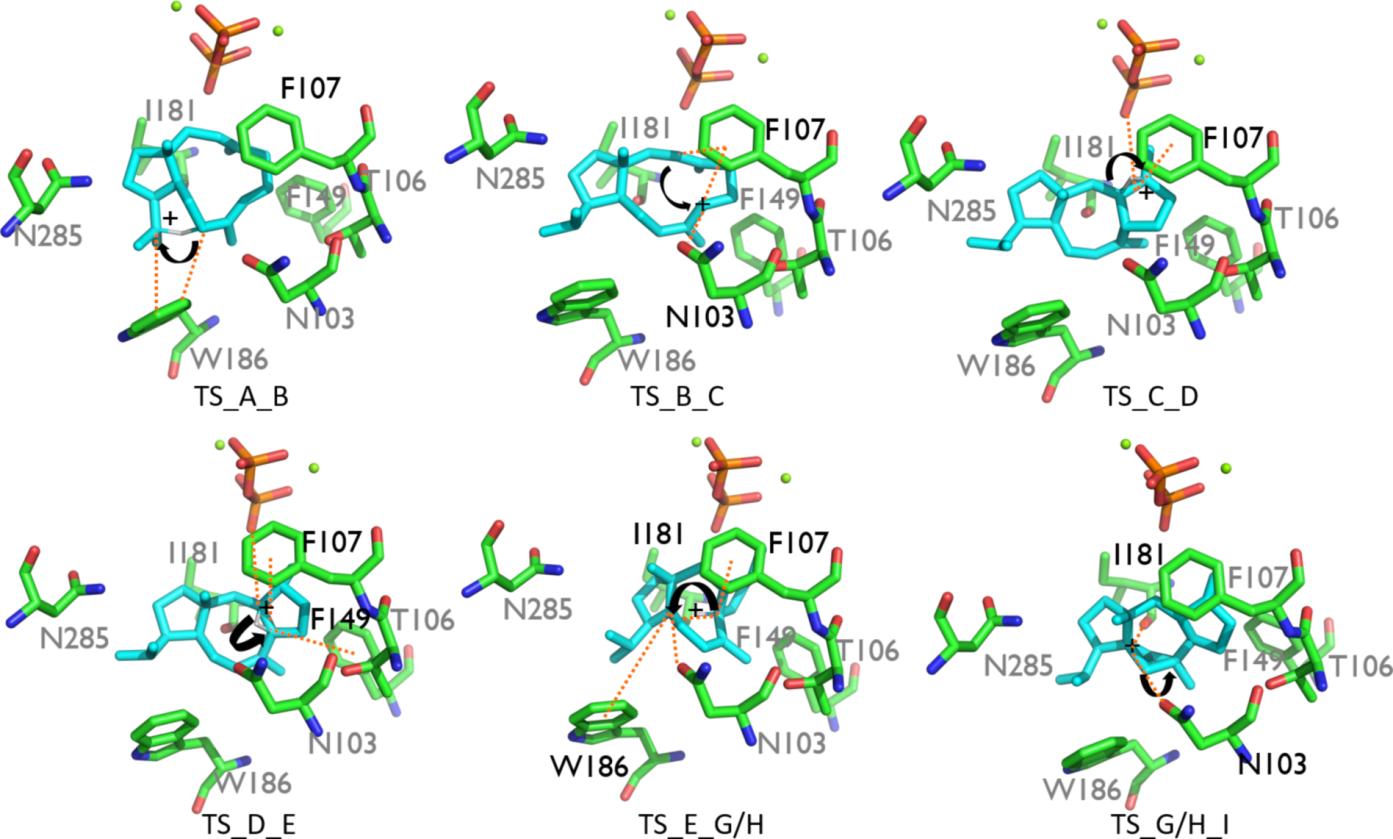
TS structures TS_**A**_**B**–TS_**G**/**H**_**I** in the active site model. Interactions are marked by dashed orange lines, the interacting residues are labeled in black, the non-interacting residues are labeled in grey, and the plus signs note the location of the cations.

**Figure 4:**
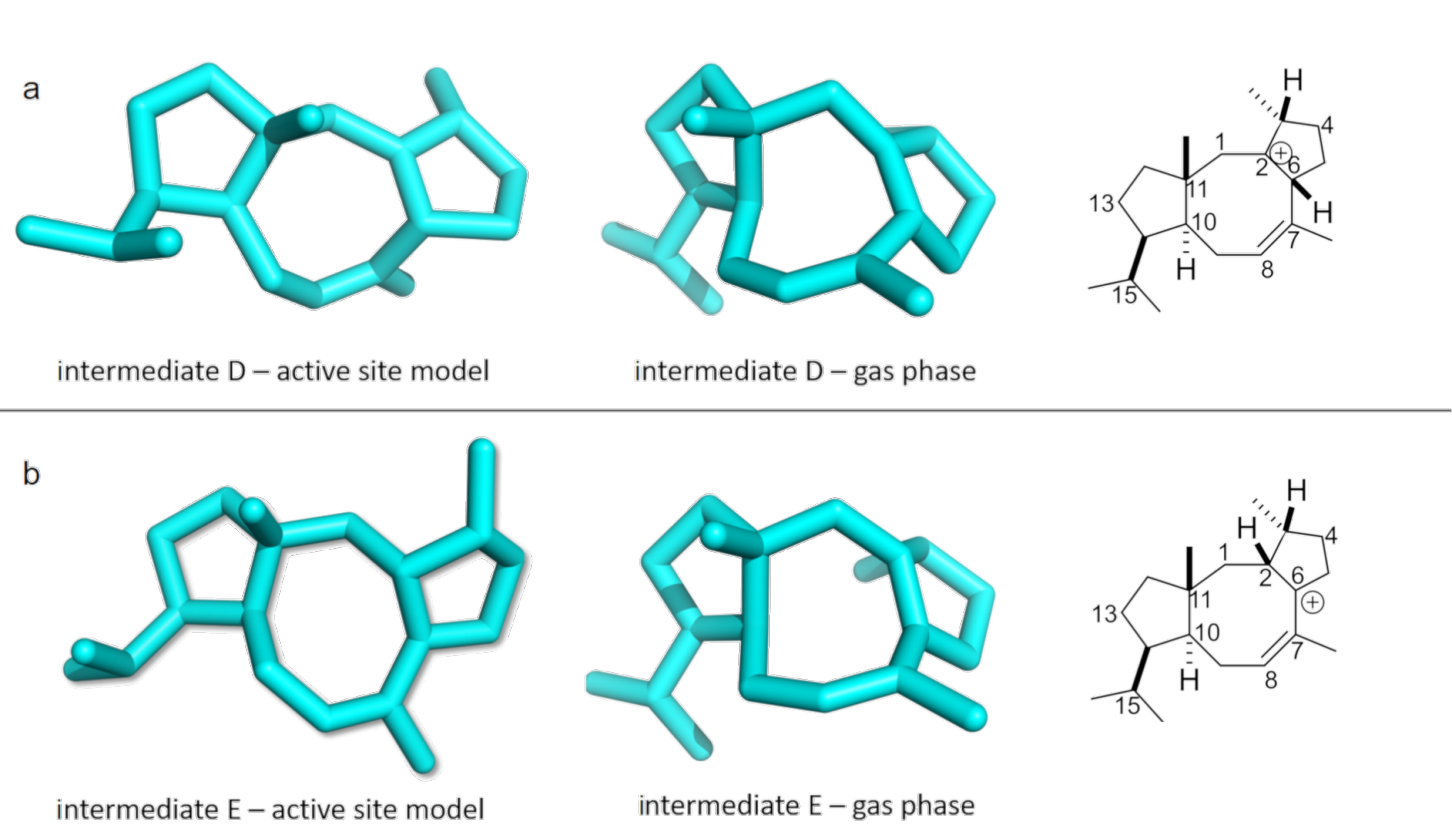
Comparison between gas phase and active site model conformations. A) Intermediate **D**. B) Intermediate **E**.

It is well established that the inherent reactivity of carbocations [27], as well as correct substrate folding in the active site [3], play crucial roles in terpene synthases. The current results highlight the importance of taking into account the active site residues while modeling terpene synthase mechanisms, as we have proposed previously [28–36,42]. We found that the energy surface in the active site model was significantly perturbed compared to the gas phase potential. Additionally, structural analysis revealed that each cation was stabilized by noncovalent interactions, such as π–cation and dipole–cation interactions. A comparison of the transition state structures in the gas phase vs the active site model is shown in Table 2. These findings suggest that the rational biosynthesis of novel terpenes might be possible by careful design of CotB2 mutants. Future studies using multiscale techniques to model the enzyme reaction in a complete enzyme environment will allow careful evaluation of the usefulness of such active site theozyme models.

**Table 2:** Comparison table of transition state structures in gas phase vs active site model.

TS structure	interaction species		gas phase	active site model
			distance (Å)	distance (Å)

TS_**A**_**B**	C_15_	H_82_	1.21	1.29
	C_8_	H_82_	1.46	1.34
	C_8_	C_15_	2.58	2.52
TS_**B**_**C**	C_2_	C_6_	2.44	2.61
TS_**C**_**D**	C_2_	H_2_	1.21	1.44
	C_3_	H_2_	1.48	1.24
	C_3_	C_2_	1.41	1.41
TS_**D**_**E**	C_6_	H_6_	1.25	1.38
	C_2_	H_6_	1.41	1.28
	C_2_	C_6_	1.41	1.42
TS_**E**_**G**/**H**	C_6_	H_6_	1.12	1.14
	C_10_	H_6_	1.74	1.63
	C_10_	C_6_	2.63	2.49
	C_8_	C_10_	2.33	2.54
TS_**H**_**I**	C_9_	C_7_	1.71	1.66
	C_9_	C_10_	1.65	1.69
	C_10_	C_7_	2.48	2.46

## Conclusion

In this work, we compared the energy profiles of the terpene cyclase CotB2 reaction obtained in the gas phase and using an active site model. The calculations used identical QM methods, facilitating a direct comparison. We presented evidence for the important role played by the active site residues in CotB2 on the reaction energetics in an active site cluster model, suggesting that reaction control in terpene synthase is obtained via a combination of inherent reactivity, initial substrate folding, and enzyme environmental effects. Specifically, the results using the active site model revealed the significant effect that the active site residues have on the relative electronic energy of the intermediates and TS structures in comparison with gas phase data due to ionic, π–cation, and dipole–cation interactions. A detailed understanding of the role of the enzyme environment on the reaction cascade in CotB2 can provide important information to derive a synthetic strategy for cyclooctatin and related terpene manufacturing. Future studies using hybrid quantum mechanics and molecular mechanics techniques to model the enzyme reaction in a complete enzyme environment will allow careful evaluation of the usefulness of such active site theozyme models.

## Experimental

All calculations were carried out with Gaussian 16 [47]. Geometry optimizations, frequency calculations, and intrinsic coordinate calculations were performed using the M062X/6-31+G(d,p) level of theory [48]. The gas phase structures were taken from Sato and co-workers [39]. The amino acid cage was constructed from six amino acids, which were located around the substrate and constituted part of the catalytic pocket of the enzyme (PDB-ID 6GGI) [42]. The chosen amino acids were the ones that we presumed stabilized the carbocations the most during the reaction. The coordinates of the amino acids and the substrate GGPP were taken from the corresponding X-ray structure, with a resolution of 1.8 Å [42]. In this approach, geometry optimizations with the “Modredundant” keyword were performed, and the active site residues, diphosphate moiety, and magnesium ions were fixed throughout the reaction progress. The entire cage system was treated using the above-mentioned DFT method. In order to find the TS structures, complete TS optimizations using the keywords "QST2", "QST3", and “Modredundant” were performed.

A main limitation of the current cluster modeling approach was freezing of the active site residues, which did not allow any accommodation of the active site to the evolving reaction intermediates. Flexible residues were not considered due to the possible perturbation of the active site contour and the rapid fluctuation of the total electronic energy as a function of amino acid residue geometry. An additional obvious limitation were medium and long-range nonbonded interactions beyond the active site cage considered here. These effects could be considerable and will be scrutinized in future work.

The Cartesian coordinates of all species are reported in Supporting Information File 1.

## Supporting Information

File 1 Cartesian coordinates for all species.
